# Seroprevalence of coronavirus disease 2019 (COVID-19) among health care workers from three pandemic hospitals of Turkey

**DOI:** 10.1371/journal.pone.0247865

**Published:** 2021-03-03

**Authors:** Gizem Alkurt, Ahmet Murt, Zeki Aydin, Ozge Tatli, Nihat Bugra Agaoglu, Arzu Irvem, Mehtap Aydin, Ridvan Karaali, Mustafa Gunes, Batuhan Yesilyurt, Hasan Turkez, Adil Mardinoglu, Mehmet Doganay, Filiz Basinoglu, Nurhan Seyahi, Gizem Dinler Doganay, Hamdi Levent Doganay

**Affiliations:** 1 Genomic Laboratory (GLAB), Umraniye Teaching and Research Hospital, University of Health Sciences, Istanbul, Turkey; 2 Cerrahpasa Faculty of Medicine, Department of Nephrology, Istanbul University-Cerrahpasa, Istanbul, Turkey; 3 Department of Nephrology, Darica Farabi Teaching and Research Hospital, Kocaeli, Turkey; 4 Department of Molecular Biology and Genetics, Istanbul Technical University, Istanbul, Turkey; 5 Department of Molecular Biology and Genetics, Istanbul Medeniyet University, Istanbul, Turkey; 6 Department of Microbiology, Umraniye Teaching and Research Hospital, University of Health Sciences, Istanbul, Turkey; 7 Department of Infectious Disease, Umraniye Teaching and Research Hospital, University of Health Sciences, Istanbul, Turkey; 8 Cerrahpasa Faculty of Medicine, Department of Infectious Disease, Istanbul University-Cerrahpasa, Istanbul, Turkey; 9 Department of Urology, Darica Farabi Teaching and Research Hospital, Kocaeli, Turkey; 10 Health Institutes of Turkey (TUSEB), Istanbul, Turkey; 11 Faculty of Medicine, Department of Medical Biology, Ataturk University, Erzurum, Turkey; 12 Science for Life Laboratory, KTH - Royal Institute of Technology, Stockholm, Sweden; 13 Faculty of Dentistry, Centre for Host-Microbiome Interactions, Oral & Craniofacial Sciences, King’s College London, London, United Kingdom; 14 Faculty of Medicine, Department of Infectious Diseases, Lokman Hekim University, Ankara, Turkey; 15 Department of Medical Biochemistry, Darica Farabi Teaching and Research Hospital, Kocaeli, Turkey; Federal University of Rio de Janeiro, BRAZIL

## Abstract

COVID-19 is a global threat with an increasing number of infections. Research on IgG seroprevalence among health care workers (HCWs) is needed to re-evaluate health policies. This study was performed in three pandemic hospitals in Istanbul and Kocaeli. Different clusters of HCWs were screened for SARS-CoV-2 infection. Seropositivity rate among participants was evaluated by chemiluminescent microparticle immunoassay. We recruited 813 non-infected and 119 PCR-confirmed infected HCWs. Of the previously undiagnosed HCWs, 22 (2.7%) were seropositive. Seropositivity rates were highest for cleaning staff (6%), physicians (4%), nurses (2.2%) and radiology technicians (1%). Non-pandemic clinic (6.4%) and ICU (4.3%) had the highest prevalence. HCWs in “high risk” group had similar seropositivity rate with “no risk” group (2.9 vs 3.5 *p* = 0.7). These findings might lead to the re-evaluation of infection control and transmission dynamics in hospitals.

## Introduction

In late 2019, a novel coronavirus (SARS-CoV-2) has emerged in Wuhan, China and posed a global threat to public health with a quick spread and escalating mortality. As of June, 2020, SARS-CoV-2 related disease COVID-19 affected more than nine million people worldwide, and caused more than one million deaths [1]. This is the third coronavirus outbreak that the world has faced in the last two decades, and it apparently will not be the last.

Typical initial clinical signs of COVID-19 have been reported as fever, dry cough, fatigue, headache and shortness of breath [2]. Less commonly, diarrhea, nausea and vomiting were also reported as the atypical symptoms of the disease [3]. Older age and comorbid conditions, particularly hypertension and diabetes increase hospitalization and mortality rates among infected individuals. Unknown percentage of asymptomatic carriers is another concern due to the difficulty of tracing their contacts with possible inaccurate calculations of R-naught [4,5].

Based on genome sequence analysis, SARS-CoV-2 genome was reported to contain 14 Open Reading Frames (ORFs) encoding 27 proteins [6]. The lipid envelope of the virus possesses primarily three structural proteins including membrane (M), envelope (E), and spike (S) proteins. Both S and N proteins have been reported as highly abundant and immunogenic, which makes them potential targets for serological diagnosis [7]. Besides the viral nucleic acid detection based on real-time reverse transcription polymerase chain reaction (real-time RT-PCR) [8], rapid tests and immunoassay tests were recently developed for accurate detection of IgG and IgM antibodies against SARS-CoV-2 in sera samples. Such tests may help to eliminate false negatives of RT-PCR tests caused by the difference in the viral load of different respiratory specimens [9]. For increased sensitivity in diagnosis of COVID-19, both modalities should be combined as complementary to each other. Although, serologic tests could also be utilized for the detection of overall infection rate in the population [10], the finding that antibody titer against SARS-CoV-2 decreases over time [11,12] would limit the use of serologic tests as a tool for such a purpose.

Istanbul is the epicenter of the ongoing pandemic in Turkey and 60% of the confirmed cases are from Istanbul [13]. In the current study, seroprevalence of COVID-19 specific IgGs was tested among health care workers (HCWs) from two different pandemic hospitals in Istanbul and one from the neighboring city of Kocaeli. First COVID-19 case was officially reported on 11 March 2020 in Turkey and since then all three hospitals had a substantial role with treating more than 40 000 patients during pandemic. We aimed to identify asymptomatic infections and to assess the risks of infection with SARS-CoV-2 among different clusters of HCWs. Evaluating the prevalence of infection at different parts of the hospitals and the effect of transmission prevention measures hold great importance for the development of mitigation strategies.

## Methods

### Study design and participants

This study was conducted in three pandemic hospitals in Istanbul and Kocaeli, including University of Health Sciences Umraniye Teaching and Research Hospital (UEAH), Istanbul University-Cerrahpasa, Cerrahpasa Medical Faculty Hospital (Cerrahpasa), Darica Farabi Teaching and Research Hospital (Farabi). HCWs were invited to participate in the study. Exposure risk of the HCWs was determined by the working areas they were assigned during the pandemic. Some HCWs were on administrative leave due to their medical conditions and they were classified as having “no risk”. HCWs working in the clinics, which were kept clear of COVID-19 or had no direct contact with any patient, were classified as having “low risk”. HCWs employed in hot zones for COVID-19 transmission including emergency unit, intensive care unit (ICU), pandemic clinics, COVID outpatient clinics, COVID testing labs, departments of Infectious Diseases and Chest Medicine, and radiology department (where CT scans and chest X-rays were performed) were classified as having “high risk”. HCWs with administrative roles for supervising hot zones with regular visits were also classified in the “high risk” group. We also recruited HCWs who were diagnosed with COVID-19 at least 14 days before enrollment for the study. This group was defined as the “PCR positive” group. Demographics data, comorbidities, drug history, date of COVID-19 diagnosis, past PCR tests, results of chest computed tomography (CT) scans were noted. HCWs not willing to give consent, HCWs with a history of COVID-19 diagnosis without a confirmatory PCR test and those diagnosed within the last 14 days were excluded. All blood samples were collected in the three hospitals in between 30^th^ May and 6^th^ of June 2020. Oro-nasopharyngeal swab samples of the three subgroups (excluding PCR positive group) were also tested to confirm the absence of active infection at the time when blood specimens were collected.

All sera samples were aliquoted after centrifugation of peripheral blood tubes at 800xg for 12 minutes and sera samples were kept in -20°C until the study day. For detection of SARS-CoV-2 IgG, chemiluminescent microparticle immunoassay (Abbott Laboratories, Cat no: 6R86, Lot no: 16253FN00) was carried out according to manufacturer’s instructions and samples were run on the related instrument (ARCHITECT, Abbott Laboratories, Abbott Park, IL, USA). Minimum 100 μL of serum was required for analysis. Qualitative results were reported by the instrument with the cut-off value of 1.40 S/C as recommended. This study was approved by the ethics committee of the Umraniye Teaching and Research Hospital (approval number: 29.05.2020/10337). Written informed consent was obtained from each enrolled participant. All study was carried out in accordance with the ethical standards of the Helsinki Declaration.

### Statistical analysis

All statistical analyses were performed by SPSS version 22 software (Chicago, IL). Parametric variables were analyzed by Student’s *t*-test. Mann-Whitney U test was employed for nonparametric continuous variables. Categorical variables were tested by using the chi-square and Fisher’s exact test. Two-sided *p* value below 0.05 was considered to be statistically significant.

In the end of May, Ministry of Health Department declared that the seropositivity in population was 0.8% in Turkey and there was no seroprevalence study for HCWs until June 2020. Also, in another study, seropositivity was 8% for HCWs in Spain [14]. Therefore, to compare the seroprevalence of COVID-19 between no risk group and high risk group with 90% power and 0.05 error rate, we calculated a minimum sample size of 109 for no risk group and 436 for high risk group with an enrollment ratio of 4:1. We recruited participants more than minimum sample size to uphold power after possible drop-outs (MedCalc Statistical Software version 19.1 (MedCalc Software bv, Ostend, Belgium; https://www.medcalc.org; 2019)).

## Results

### Demographic data

**Fig 1:**
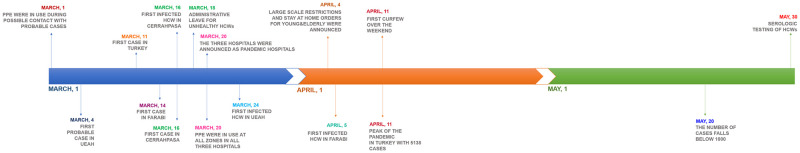
Timeline of COVID19 in three pandemic hospitals.

The timeline showing the progression of COVID-19 pandemic in Turkey, which includes events from the date that the country’s first coronavirus case has been announced, was given in Fig 1. All three pandemic hospitals are tertiary health care centers with high capacity, employing a total of 8328 HCWs. On the peak day of the pandemic, maximum hospitalized patient numbers (on a day) reached to 410, 222 and 300 for UEAH, Cerrahpasa and Farabi, respectively. Total number of hospitalized patients in the hospitals throughout the pandemic was 5437 (Table 1). Among 8328 HCWs, 932 were enrolled for the study. Demographics, assigned work areas during pandemic, comorbidities and SARS-CoV-2 IgG positivity according to risk stratification of HCWs were given on Table 2. Of all participants, the number of personnel classified as “no risk”, “low risk” and “high risk” group were 113 (12.1%), 157 (16.8%) and 543 (58.3%), respectively. Additional 119 HCWs (12.8% of all participants), previously diagnosed with COVID-19 by RT-PCR, were enrolled and classified as “PCR positive” group. Of PCR positive HCWs, 103 displayed the clinical signs of COVID-19. 16 (13.4%), who did not have compatible symptoms, were tested due to the exposure history, and were recorded as asymptomatic. Only one HCW had a history of admission to ICU, no deaths occurred (Table 1). Of the non-infected HCWs, 597 gave consent for oro-nasopharyngeal swab sampling and all RT-PCR results were negative.

**Table 1:** Characteristics of the health care facilities.

	Hospitals			
	UEAH	Cerrahpasa	Farabi	Total
**Number of Hospital Beds**	836	880	350	2066
**Maximum Number of Hospitalized Patient with COVID-19 (at the peak day)**	410	222	300	932
**Total Number of Hospitalized Patient with COVID-19**	2528	1056	1853	5437
**Total Number of Patients Visits at COVID-19 Outpatient Clinic**	14906	21970	12375	49251
**Number of HCWs**	3232	3723	1373	8328
**HCWs diagnosed with COVID-19**	125 (3.8%)	107 (2.8%)	14 (1%)	241 (2.8%)
**PCR Positive HCWs**	105 (3.2%)	84 (2.2%)	8 (0.5%)	187 (2.2%)
**Hospitalized HCWs in ICU**	1	0	0	1
**Number of Deaths in infected HCWs**	0	0	0	0

All data is given for the period between 1 March and 30 May 2020. HCW: Health Care Worker.

**Table 2:** Demographics and seropositivity of front-line and non-front-line HCWs.

	Health Care Workers				
Characteristics	No Risk n = 113 (%)	Low Risk n = 157 (%)	High Risk n = 543 (%)	PCR Positive n = 119 (%)	Total n = 932 (%)
**Hospital**					
UEAH	52 (46.0)	0	218 (40.1)	52 (43.7)	322 (34.5)
Cerrahpasa	58 (51.3)	54 (34.4)	124 (22.8)	64 (53.8)	300 (32.2)
Farabi	3 (2.7)	103 (65.6)	201 (37.0)	3 (2.5)	310 (33.3)
**Age,** *Mean± SD*	34.5± 12.23	35.5± 8.86	34.3± 8.92	36.2± 10.14	34.8± 9.54
**Sex**					
Male	56 (49.6)	53 (33.8)	184 (33.9)	39 (32.8)	332 (35.6)
Female	57 (50.4)	104 (66.2)	359 (66.1)	80 (67.2)	600 (64.4)
**Profession**					
Physician	62 (54.9)	12 (7.6)	175 (32.2)	30 (25.2)	279 (29.9)
Nurse	17 (15.0)	32 (20.4)	174 (32.0)	45 (37.8)	268 (28.8)
Ward Clerk&Security	12 (10.6)	67 (42.7)	35 (6.4)	15 (12.6)	129 (13.8)
Lab&Radiology Technician	1 (0.9)	7 (4.5)	92 (16.9)	7 (5.9)	107 (11.5)
Cleaning Staff	12 (10.6)	10 (6.4)	62 (11.4)	12 (10.1)	96 (10.3)
Administrative Staff	9 (8.0)	29 (18.5)	5 (0.9)	10 (8.4)	53 (5.7)
**Assigned Department**					
Pandemic Clinic	0	0	284 (52.3)	51 (42.8)	335 (35.9)
Intensive Care Unit	0	0	69 (12.7)	10 (8.4)	79 (8.5)
Emergency Room	0	0	47 (8.7)	8 (6.7)	55 (5.9)
Corona Lab	0	0	61 (11.2)	1 (0.8)	62 (6.7)
Swab Team	0	0	51 (9.4)	4 (3.4)	55 (5.9)
Non-Pandemic Clinic	0	50 (31.9)	28 (5.1)	30 (25.2)	108 (11.6)
Administrative Office	0	107 (68.2)	3 (0.6)	15 (12.6)	125 (13.4)
On leave	113 (100)	0	0	0	113 (12.1)
**Clinical Information**					
Chronic Disease Present	31 (27.4)	19 (12.1)	61 (11.2)	31 (26.1)	142 (15.2)
Hypertension	8 (7.1)	7 (4.5)	19 (3.5)	7 (5.9)	41 (4.4)
Diabetes Mellitus	5 (4.4)	2 (1.3)	16 (2.9)	6 (5.0)	29 (3.1)
Other Chronic Disease	22 (19.5)	11 (7.0)	29 (5.3)	19 (16.0)	81 (8.7)
ACE inh./ARB	6 (5.3)	5 (3.2)	16 (2.9)	8 (6.7)	35 (3.8)
Immuno-suppressive drug	2 (1.8)	1 (0.6)	2 (0.4)	2 (1.7)	7 (0.8)
Smoking	25 (22.1)	42 (26.8)	147 (27.1)	14 (11.8)	228 (24.5)
**IgG Positive**	4 (3.5)	2 (1.3)	16 (2.9)	93 (78.2)	115 (12.3)
Symptoms	n.a.	n.a.	n.a.	98 (82.4)	98 (10.5)
CT Result	n.a.	n.a.	n.a.	71 (59.7)	71 (7.6)

ACE inh.: Angiotensin-converting enzyme (ACE) inhibitor, ARB: Angiotensin II receptor blocker, CT: Computed tomography scan, HCW: Health Care Worker, n.a: Not applicable.

### Seroprevalence of HCWs in non-infected group

**Fig 2:**
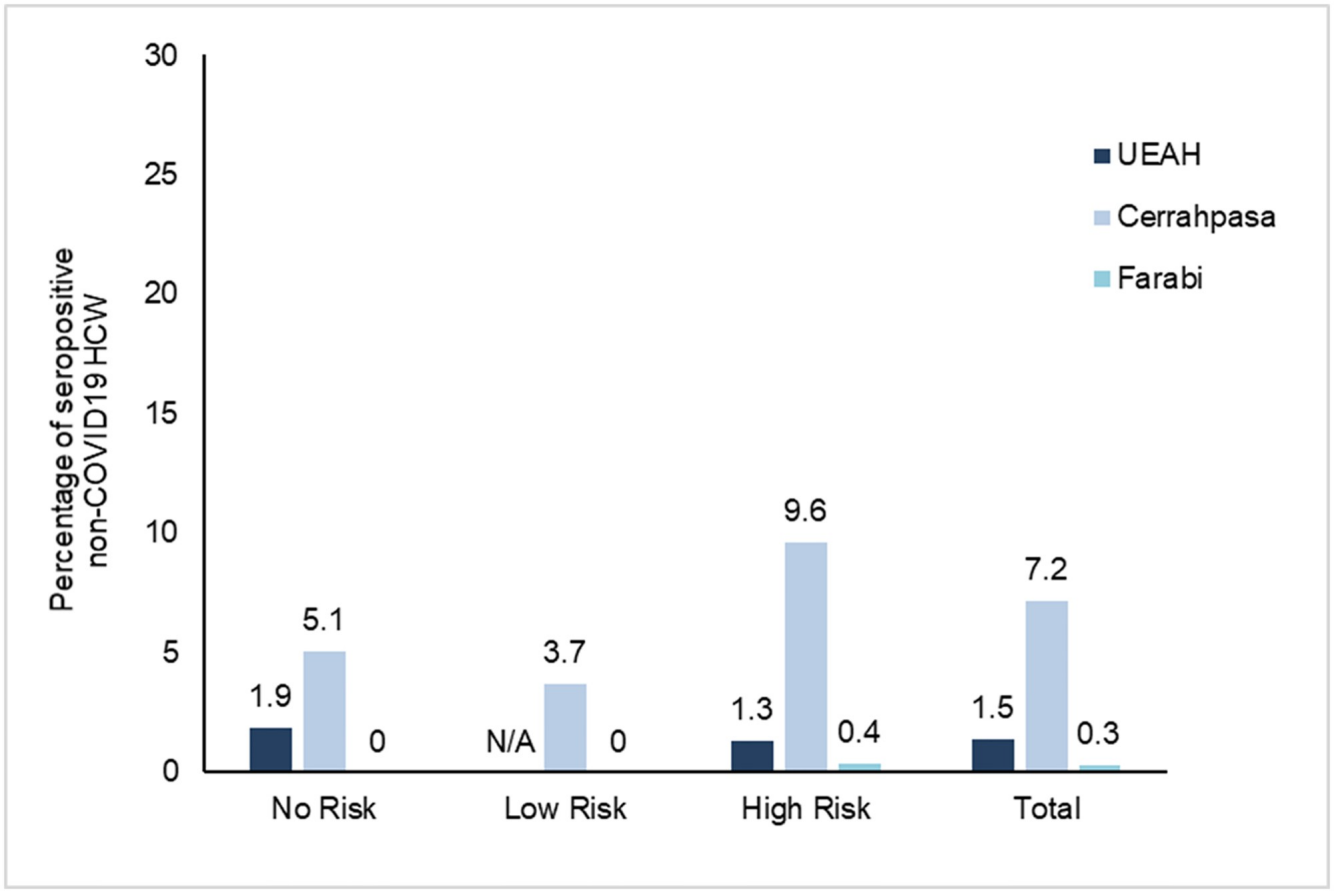
Seropositivity results of non-infected HCW in three pandemic hospitals.

**Fig 3:**
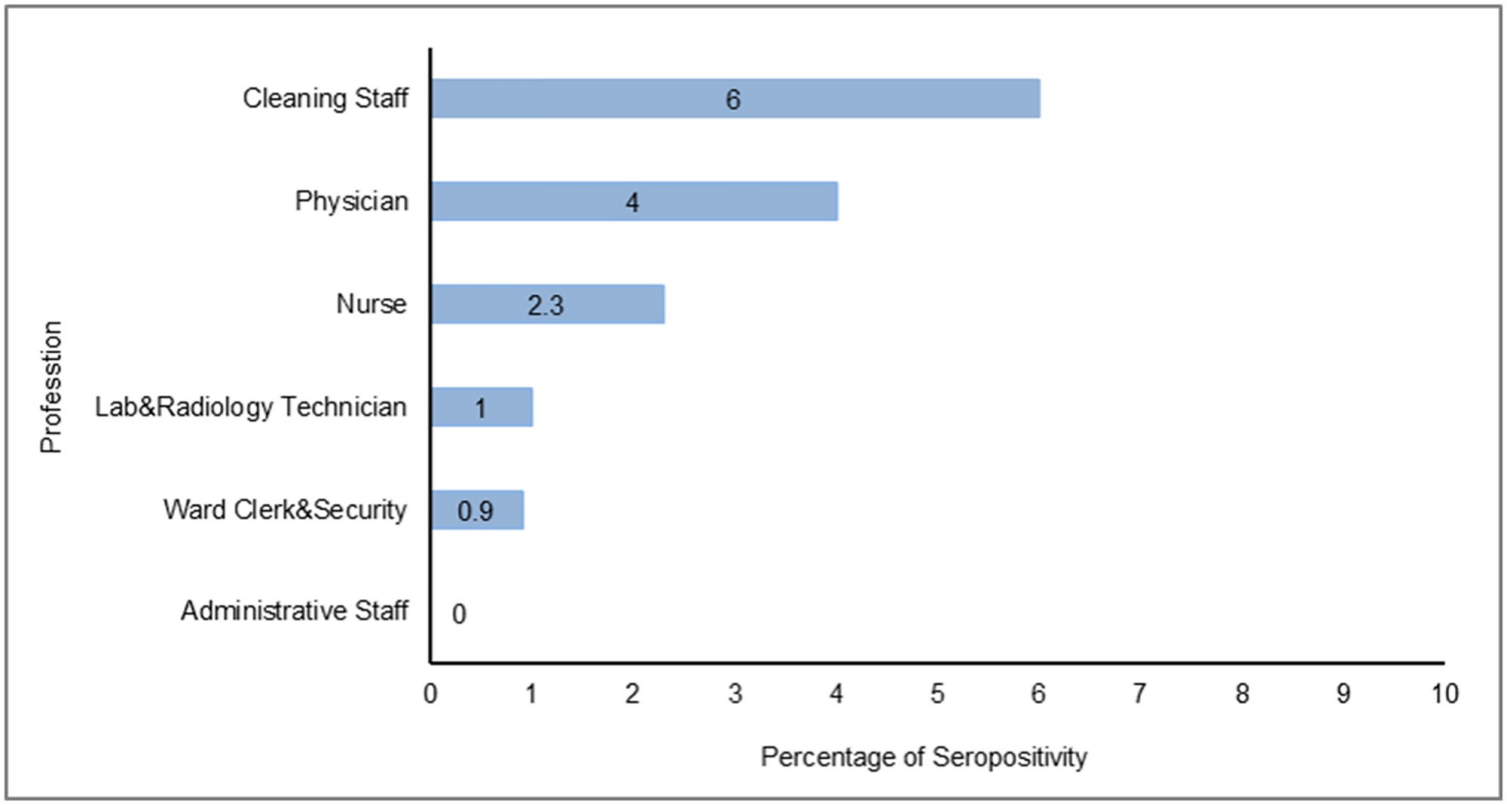
Seropositivity results of non-infected HCW according to profession.

**Fig 4:**
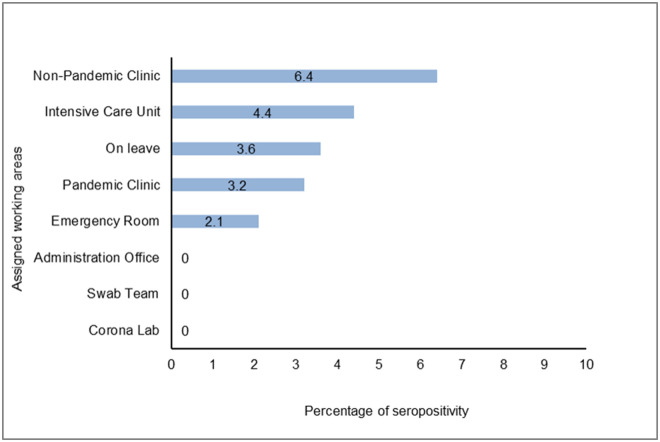
Seropositivity results of non-infected HCW according to assigned working areas.

IgG antibodies against SARS-CoV-2 in serum samples of all participants were detected by chemiluminescent microparticle immunoassay. The rate of seroprevalence was 2.7% among non-infected HCWs (S2 Table). The seropositivity rate was 3.5%, 1.3%, 2.9% in “no risk”, “low risk” and “high risk” groups, respectively (*p* = 0.4). Among three hospitals, Cerrahpasa had the highest seroprevalence (7.2%, *p*<0.001) (Table 3 and S1 Fig). The seropositivity rate in Cerrahpasa was statistically significantly different when compared to UEAH (*p*<0.01) and Farabi (*p*<0.001). Of 307 HCWs in Farabi, only one tested positive, yielding the lowest seropositivity rate of the hospitals (0.3%) (Fig 2). Although the seroprevalence was not significantly different among professions, it was highest among cleaning staff (6%) (Fig 3). Interestingly, the seropositivity rate of the workers employed in non-pandemic clinics (6.4%) was higher than those working in other areas (*p* = 0.05) (Table 4 and Fig 4).

**Table 3:** Seropositivity among risk groups.

Characteristic	No Risk n = 113 (%)	Low Risk n = 157 (%)	High Risk n = 543 (%)	Total n = 813 (%)
**IgG Positive**	4 (3.5)	2 (1.3)	16(2.9)	22 (2.7)
**Hospital**				
UEAH	1 (1.9)	N/A	3 (1.3)	4 (1.5)
Cerrahpasa	3 (5.1)	2 (3.7)	12 (9.6)	17 (7.2)
Farabi	0	0	1 (0.4)	1 (0.3)
**IgG Titration,** *mean±SD*	0.4 ±0.18	0.3 ±0.11	0.1 ±0.16	0.3 ±0.16

N/A: Not Available.

**Table 4:** Seropositivity among assigned working areas and profession.

Characteristic n (%)	IgG Negative n = 791	IgG Positive n = 22	OR	95% CI	*p* value
**Profession**					
Physician	239 (96.0)	10 (4.0)	1.93	0.82–4.51	0.16
Nurse	218 (97.7)	5 (2.2)	0.77	0.28–2.12	0.81
Ward Clerk&Security	113 (99.1)	1 (0.9)	0.29	0.04–2.15	0.35
Lab&Radiology Technician	99 (99.0)	1 (1.0)	0.33	0.04–2.50	0.51
Cleaning Staff	79 (94.0)	5 (6.0)	2.65	0.95–7.38	0.07
Administrative Staff	43 (100)	0	n.a.	n.a.	0.62
**Assigned Department**					
Pandemic Clinic	276 (96.8)	9 (3.2)	1.3	0.55–3.08	0.65
Intensive Care Unit	66 (95.6)	3 (4.3)	1.73	0.5–6.01	0.42
Emergency Room	46 (97.9)	1 (2.1)	0.77	0.10–5.86	1.00
Corona Lab	61 (100)	0	n.a.	n.a.	0.4
Swab Team	51 (100)	0	n.a.	n.a.	0.63
Non-Pandemic Clinic	73 (93.6)	5 (6.4)	2.89	1.04–8.61	0.05
Administration Office	111 (100)	0	n.a.	n.a.	0.06
On leave	107 (96.4)	4 (3.6)	1.39	0.46–4.19	0.53

n.a: Not applicable, OR: Odd Ratio, CI: Confidence Interval.

### Seroprevalence of PCR positive group

To assess antibody production in COVID-19 patients, we analyzed the positive rates of IgGs in sera of all HCWs after 52.8±11.6 days post-infection. IgGs for SARS-CoV-2 were detected in 78.2% of convalescent COVID-19 patients (S1 Table). Among PCR positive group, those had CT findings compatible with COVID-19, had higher seropositivity (*p*<0.001). IgG titers in asymptomatic PCR positive patients were significantly lower than symptomatic ones (*p* = 0.008).

## Discussion

Health care systems are under tremendous pressure due to the lack of curative treatment for COVID-19 [15]. Protection of first-line HCWs from the infection is of utmost importance to provide sustainable public health services. High burden of COVID-19 disease in hospitals worldwide has been explored in several studies. In Spain, one of the European countries hardest-hit by COVID-19, the nationwide seroprevalence for SARS-CoV-2 tested by chemiluminescent microparticle immunoassay was found to be around 5%. For HCWs, the seroprevalence has surpassed the national rate and reached over 8% [14]. Of HCWs in a tertiary hospital in Belgium, seroprevalence detected by rapid cassette test was 6.4% [16]. In this study, even though the percentage of HCWs infected with COVID-19 in the three pandemic hospitals is also noteworthy (2.7%), the insignificant difference between no-risk and high-risk group implied that the protection measures are reassuringly rigorous to prevent the transmission of SARS-CoV-2 in these hospitals.

HCWs employed in coronavirus testing labs (Corona lab) and swab teams were reasonably anticipated to be at high risk. In this study, we did not observe seropositivity in these personnel, they were found to be efficiently protected from the disease. Although the seropositivity was statistically higher in non-pandemic clinics, factors including public transportation, poor housing conditions, limited personal space and thus reduced compliance with social distancing, which are not addressed in this study, must be considered to analyze the transmission of disease among HCWs. All staff in hospitals should be well-trained on elements of disease transmission; such as the sources of exposure to the virus, risks associated with the exposure and suitable occupational protocols. Such data implied that the risk of viral transmission in these areas are widely underestimated and utmost caution is urged in all zones of the hospitals.

Limited data are available for asymptomatic or subclinical infections in transmission of SARS-CoV-2 virus [17]. Here, 13.4% of the PCR-confirmed HCWs with infection never developed any COVID-19 relevant symptoms and remained asymptomatic. Besides, a substantial number of undiagnosed HCWs were seropositive indicating the recovery from COVID-19 without any clinical signs of the disease. These asymptomatic carriers who remained undiagnosed throughout the infection may be the silent sources of virus spread among HCWs. The finding that IgG levels of asymptomatic individuals were lower than that of symptomatic ones is in line with the previous findings suggesting that asymptomatic carriers have a weaker humoral immune response to COVID-19 infection [18]. Similarly, seropositivity rate was statistically significantly higher in those with compatible CT scan findings, indicating that the severity of the disease is positively correlated with the potency of immunity. Such a finding is similar to that were reported for SARS-CoV and MERS-CoV, for whom higher levels of IgM and IgGs have also been shown to be correlated with the severity of disease [19–22].

Although overall seropositivity among HCWs was calculated as 2.7% in our study, fluctuations between institutions were also noted. A lower rate at Farabi (0.3%) might be presumable, as PCR confirmed HCW rate was also not that high (0.5%). On the other hand, between two institutions with comparable PCR confirmed HCW rates, observed seropositivity of HCWs from Cerrahpasa was found to be significantly higher than those from UEAH (7.2% and 1.5% respectively). As we compared the work schedules of HCWs in three hospitals, we noticed that physicians in Cerrahpasa was assigned to the pandemic clinics on daily basis. On the other days of the week the same physician served at non-pandemic clinic, too. The work schedule was made on monthly basis in UEAH and Farabi. Moreover, at Cerrahpasa as being a distinguished medical school, teaching on ward rounds with medical students resumed until 18^th^ of March. UEAH and Farabi do not teach undergraduate students. Cerrahpasa’s buildings were being reconstructed when the outbreak began in Turkey and this might have also impaired some infection control measures.

The information for SARS-CoV-2 transmission among health care workers could help for the revision of health policies and immunization strategies in hospitals for a possible resurgence of the outbreak. Further, extensive knowledge of antibody seroconversion and characterization of antibody profiles throughout SARS-CoV-2 infection could provide insights for the identification of potentially targeted neutralizing antibodies.

### Limitations

Even though a significant number of employees were tested, not all of the invited HCWs in these hospitals participated in the study. Screening larger cohorts from hospitals could serve more information to monitor the course of pandemic among HCWs. Furthermore, a group of HCWs might have be reported as negative just because of the tendency of IgG titers to drop time-dependently.

## Supporting information

S1 Fig Result of IgG titration means according to risk groups. (DOCX)

S1 Table IgG titration values of “PCR positive” group. (DOCX)

S2 Table IgG titration values of seropositive non-infected HCWs. (DOCX)

## References

[1] WHO Coronavirus Disease (COVID-19) Dashboard | WHO Coronavirus Disease (COVID-19) Dashboard. [cited 3 Jul 2020]. https://covid19.who.int/.

[2] Guan W, Ni Z, Hu Y, Liang W, Ou C, He J, et al. Clinical Characteristics of Coronavirus Disease 2019 in China. N Engl J Med. 2020;382: 1708–1720. 10.1056/NEJMoa2002032 32109013 PMC7092819

[3] Wang J, Zhou M, Liu F. Reasons for healthcare workers becoming infected with novel coronavirus disease 2019 (COVID-19) in China. Journal of Hospital Infection. W.B. Saunders Ltd; 2020. pp. 100–101. 10.1016/j.jhin.2020.03.002 32147406 PMC7134479

[4] Park SW, Cornforth DM, Dushoff J, Weitz JS. The time scale of asymptomatic transmission affects estimates of epidemic potential in the COVID-19 outbreak. medRxiv. 2020; 2020.03.09.20033514. 10.1101/2020.03.09.20033514 32446187 PMC7212980

[5] Yu X, Yang R. COVID-19 transmission through asymptomatic carriers is a challenge to containment. Influenza and other Respiratory Viruses. Blackwell Publishing Ltd; 2020. p. 474. 10.1111/irv.12743 32246886 PMC7228388

[6] Wu A, Peng Y, Huang B, Ding X, Wang X, Niu P, et al. Genome Composition and Divergence of the Novel Coronavirus (2019-nCoV) Originating in China. Cell Host Microbe. 2020;27: 325–328. 10.1016/j.chom.2020.02.001 32035028 PMC7154514

[7] Okba NMA, Müller MA, Li W, Wang C, GeurtsvanKessel CH, Corman VM, et al. Severe Acute Respiratory Syndrome Coronavirus 2-Specific Antibody Responses in Coronavirus Disease 2019 Patients. Emerg Infect Dis. 2020;26. 10.3201/eid2607.200841 32267220 PMC7323511

[8] Shih HI, Wu CJ, Tu YF, Chi CY. Fighting COVID-19: A quick review of diagnoses, therapies, and vaccines. Biomedical Journal. Elsevier B.V.; 2020. 10.1016/j.bj.2020.05.021 32532623 PMC7260535

[9] Liu W, Liu L, Kou G, Zheng Y, Ding Y, Ni W, et al. Evaluation of Nucleocapsid and Spike Protein-Based Enzyme-Linked Immunosorbent Assays for Detecting Antibodies against SARS-CoV-2. J Clin Microbiol. 2020;58. 10.1128/JCM.00461-20 32229605 PMC7269413

[10] Lou B, Li T-D, Zheng S-F, Su Y-Y, Li Z-Y, Liu W, et al. Serology characteristics of SARS-CoV-2 infection since the exposure and post symptoms onset. medRxiv. 2020; 2020.03.23.20041707. 10.1101/2020.03.23.20041707 PMC7401320 32430429

[11] Bruni M, Cecatiello V, Diaz-Basabe A, Lattanzi G, Mileti E, Monzani S, et al. Persistence of Anti-SARS-CoV-2 Antibodies in Non-Hospitalized COVID-19 Convalescent Health Care Workers. J Clin Med. 2020;9: 3188. 10.3390/jcm9103188 33019628 PMC7600936

[12] Long QX, Tang XJ, Shi QL, Li Q, Deng HJ, Yuan J, et al. Clinical and immunological assessment of asymptomatic SARS-CoV-2 infections. Nat Med. 2020;26: 1200–1204. 10.1038/s41591-020-0965-6 32555424

[13] Doganay L. Responding to Covid-19 in Istanbul: Perspective from Genomic Laboratory. North Clin Istanbul. 2020;7: 311. 10.14744/nci.2020.30075 32478308 PMC7251262

[14] Pollán M, Pérez-Gómez B, Pastor-Barriuso R, Oteo J, Hernán MA, Pérez-Olmeda M, et al. Prevalence of SARS-CoV-2 in Spain (ENE-COVID): a nationwide, population-based seroepidemiological study. Lancet. 2020;0. 10.1016/S0140-6736(20)31483-5 32645347 PMC7336131

[15] Wang D, Hu B, Hu C, Zhu F, Liu X, Zhang J, et al. Clinical Characteristics of 138 Hospitalized Patients with 2019 Novel Coronavirus-Infected Pneumonia in Wuhan, China. JAMA—J Am Med Assoc. 2020;323: 1061–1069. 10.1001/jama.2020.1585 32031570 PMC7042881

[16] Steensels D, Oris E, Coninx L, Nuyens D, Delforge M-L, Vermeersch P, et al. Hospital-Wide SARS-CoV-2 Antibody Screening in 3056 Staff in a Tertiary Center in Belgium. JAMA. 2020 [cited 3 Jul 2020]. 10.1001/jama.2020.11160 32539107 PMC7296458

[17] Weitz JS, Beckett SJ, Coenen AR, Demory D, Dominguez-Mirazo M, Dushoff J, et al. Modeling shield immunity to reduce COVID-19 epidemic spread. Nat Med. 2020;26: 849–854. 10.1038/s41591-020-0895-3 32382154 PMC8272982

[18] Long Q-X, Tang X-J, Shi Q-L, Li Q, Deng H-J, Yuan J, et al. Clinical and immunological assessment of asymptomatic SARS-CoV-2 infections. Nat Med. 2020; 1–5. 10.1038/s41591-019-0740-8 32555424

[19] Lee N, Chan PKS, Ip M, Wong E, Ho J, Ho C, et al. Anti-SARS-CoV IgG response in relation to disease severity of severe acute respiratory syndrome. J Clin Virol. 2006;35: 179–184. 10.1016/j.jcv.2005.07.005 16112612 PMC7108264

[20] Alshukairi AN, Khalid I, Ahmed WA, Dada AM, Bayumi DT, Malic LS, et al. Antibody response and disease severity in healthcare worker MERS survivors. Emerg Infect Dis. 2016;22: 1113–1115. 10.3201/eid2206.160010 27192543 PMC4880093

[21] Zhang B, Zhou X, Zhu C, Feng F, Qiu Y, Feng J, et al. Immune phenotyping based on neutrophil-to-lymphocyte ratio and IgG predicts disease severity and outcome for patients with COVID-19. medRxiv. 2020; 2020.03.12.20035048. 10.3389/fmolb.2020.00157 32719810 PMC7350507

[22] Zhao J, Yuan Q, Wang H, Liu W, Liao X, Su Y, et al. Antibody responses to SARS-CoV-2 in patients of novel coronavirus disease 2019. medRxiv. 2020. 10.1093/cid/ciaa344 32221519 PMC7184337

