# Supplementary material for: Seroprevalence of coronavirus disease 2019 (COVID-19) among health care workers from three pandemic hospitals of Turkey
Source: PLoS One. 2021 Mar 3;16(3):e0247865. doi: 10.1371/journal.pone.0247865 (PMC7928442; doi:10.1371/journal.pone.0247865)

**Seroprevalence of Coronavirus Disease 2019 (COVID-19) among health care workers from three pandemic hospitals of Turkey**

**Gizem ALKURT^1¶^, Ahmet MURT^2¶^, Zeki AYDIN^3¶^, Ozge TATLI^4¶^, Nihat Bugra AGAOGLU^1^, Arzu IRVEM^5^, Mehtap AYDIN^6^, Ridvan KARAALI^7^, Mustafa GUNES^8^, Batuhan YESILYURT^9^, Hasan TURKEZ^10^, Adil MARDINOGLU^11,12^, Mehmet DOGANAY^13^, Filiz BASINOGLU^14^*, Nurhan SEYAHI^2^*, Gizem DINLER DOGANAY^4^*, Levent DOGANAY^1^***

^1^ Genomic Laboratory (GLAB), Umraniye Teaching and Research Hospital, University of Health Sciences, Istanbul, Turkey

^2^ Department of Nephrology, Cerrahpasa Faculty of Medicine, Istanbul University-Cerrahpasa, Istanbul, Turkey

^3^ Department of Nephrology, Darica Farabi Teaching and Research Hospital, Kocaeli, Turkey

^4^ Department of Molecular Biology and Genetics, Istanbul Technical University, Istanbul, Turkey

^5^ Department of Microbiology, Umraniye Teaching and Research Hospital, University of Health Sciences, Istanbul, Turkey

^6^ Department of Infectious Disease, Umraniye Teaching and Research Hospital, University of Health Sciences, Istanbul, Turkey

^7^ Department of Infectious Disease, Cerrahpasa Faculty of Medicine, Istanbul University-Cerrahpasa, Istanbul, Turkey

^8^ Department of Urology, Darica Farabi Teaching and Research Hospital, Kocaeli, Turkey

^9^ Health Institutes of Turkey (TUSEB), Istanbul, Turkey

^10^ Department of Medical Biology, Faculty of Medicine, Atatürk University, Erzurum, Turkey

^11^ Science for Life Laboratory, KTH - Royal Institute of Technology, Stockholm, Sweden

^12^ Centre for Host-Microbiome Interactions, Faculty of Dentistry, Oral & Craniofacial Sciences, King’s College London, London, United Kingdom

^13^ Department of Infectious Diseases, Erciyes University, Kayseri, Turkey

^14^ Department of Medical Biochemistry, Darica Farabi Teaching and Research Hospital, Kocaeli, Turkey

***Corresponding authors:**

Levent DOGANAY, MD. E-mail: [levent.doganay@saglik.gov.tr](mailto:levent.doganay@saglik.gov.tr) (LD)

Gizem DINLER DOGANAY, PhD. E-mail: gddoganay@itu.edu.tr (GDD)

Nurhan SEYAHI, MD. E-mail: [nseyahi@yahoo.com](mailto:nseyahi@yahoo.com) (NS)

Filiz BASINOGLU, MD. E-mail: [fbasinoglu@yahoo.com](mailto:fbasinoglu@yahoo.com) (FB)

**¶ These authors contributed to the study equally.**

***** **These authors have equal correspondence.**

**SUPPLEMENTARY DATA**

B

A

**S1 Fig: Result of IgG titration means according to risk groups.** A) No, low and high risk group HCWs’ IgG titration B) No, low and high risk group seropositive HCWs’ IgG titration


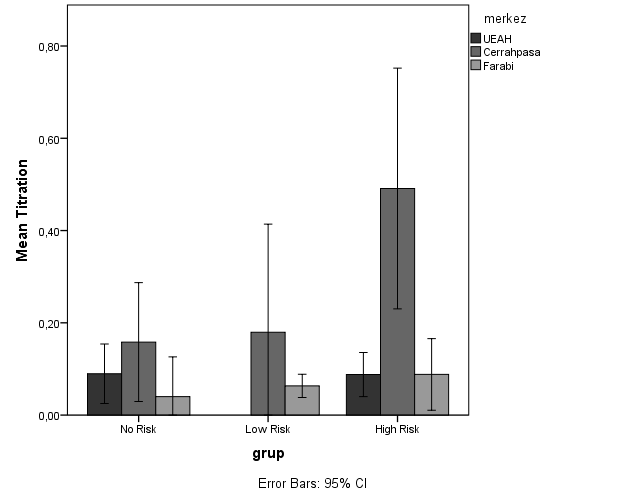

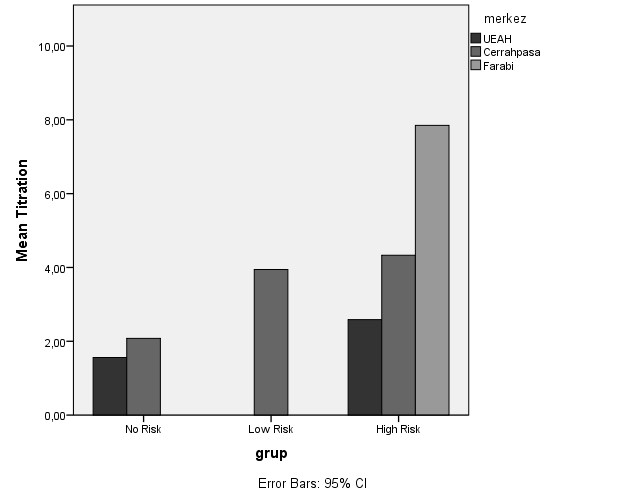

Supplement: S1 Fig — (DOCX) [file pone.0247865.s001.docx]
