# Supplementary material for: Seroprevalence of coronavirus disease 2019 (COVID-19) among health care workers from three pandemic hospitals of Turkey
Source: PLoS One. 2021 Mar 3;16(3):e0247865. doi: 10.1371/journal.pone.0247865 (PMC7928442; doi:10.1371/journal.pone.0247865)
Supplement: S1 Table — (DOCX) [file pone.0247865.s002.docx]

**S1 Table: IgG titration values of “PCR positive” group**

| **Patient Number** | **IgG Titration (S/C)** | **Patient Number** | **IgG Titration**  **(S/C)** |
| --- | --- | --- | --- |
| C-255 | 8·51 | UP-2 | 6·03 |
| C-220 | 7·92 | UP-52 | 6·03 |
| UP-15 | 7·88 | UP-8 | 5·90 |
| C-231 | 7·88 | UP-49 | 5·87 |
| UP-53 | 7·68 | UP-17 | 5·79 |
| C-156 | 7·58 | C-63 | 5·76 |
| UP-18 | 7·05 | C-164 | 5·71 |
| UP-37 | 6·97 | C-136 | 5·44 |
| C-214 | 6·80 | UP-9 | 5·43 |
| C-292 | 6·78 | C-73 | 5·42 |
| C-289 | 6·65 | C-99 | 5·41 |
| C-225 | 6·64 | C-204 | 5·41 |
| C-241 | 6·56 | UP-13 | 5·41 |
| C-242 | 6·43 | C-228 | 5·31 |
| C-81 | 6·41 | C-247 | 5·28 |
| UP-26 | 6·38 | C-134 | 5·25 |
| UP-50 | 6·25 | UP-31 | 5·09 |
| C-100 | 6·18 | C-7 | 4·83 |
| UP-10 | 6·15 | C-298 | 4·77 |
| C-184 | 6·14 | UP-3 | 4·67 |
| C-296 | 6·11 | C-70 | 4·64 |
| UP-44 | 6·05 | C-52 | 4·61 |
| C-131 | 6·04 | UP-30 | 4·57 |
| UP-32 | 4·56 | UP-11 | 3·80 |
| C-113 | 4·32 | C-65 | 3·80 |
| UP-45 | 4·32 | UP-12 | 3·78 |
| UR-152 | 4·30 | UP-7 | 3·67 |
| C-119 | 4·27 | UP-22 | 3·66 |
| UP-29 | 4·26 | UR-155 | 3·65 |
| C-183 | 4·25 | C-96 | 3·54 |
| C-277 | 4·21 | C-69 | 3·51 |
| C-144 | 4·18 | UP-42 | 1·67 |
| UP-38 | 4·17 | C-179 | 1·67 |
| C-229 | 3·99 | UR-153 | 1·50 |
| C-249 | 3·97 | UP-6 | 1·41 |
| UP-54 | 3·81 |  |  |
|  |  |  |  |
