# Supplementary material for: Seroprevalence of coronavirus disease 2019 (COVID-19) among health care workers from three pandemic hospitals of Turkey
Source: PLoS One. 2021 Mar 3;16(3):e0247865. doi: 10.1371/journal.pone.0247865 (PMC7928442; doi:10.1371/journal.pone.0247865)
Supplement: S2 Table — (DOCX) [file pone.0247865.s003.docx]

**S2 Table: IgG titration values of seropositive non-infected HCWs**

| **Patient Number** | **IgG Titration (S/C)** | **Patient Number** | **IgG Titration**  **(S/C)** |
| --- | --- | --- | --- |
| C-273 | 7·89 | C-172 | 2·68 |
| D-53 | 7·85 | C-13 | 2·42 |
| C-264 | 7·56 | C-15 | 2·35 |
| C-299 | 6·29 | C-216 | 2·05 |
| C-246 | 6·18 | C-104 | 1·92 |
| C-55 | 6·11 | C-282 | 1·78 |
| C-88 | 5·23 | UR-48 | 1·66 |
| C-64 | 4·63 | C-114 | 1·64 |
| C-240 | 4·55 | UR-223 | 1·56 |
| UR-173 | 4·54 | UT-15 | 1·56 |
| C-251 | 2·79 | C-265 | 1·41 |
